# Economic Evaluation of Lifestyle Interventions for Preventing Diabetes and Cardiovascular Diseases

**DOI:** 10.3390/ijerph7083150

**Published:** 2010-08-09

**Authors:** Sanjib Saha, Ulf-G Gerdtham, Pia Johansson

**Affiliations:** 1 Centre for Primary Health Care Research, Lund University, Lund, Sweden; 2 Health Economics & Management, Institute of Economic Research, Lund University, Lund, Sweden; 3 Department of Economics, Lund University, Lund, Sweden; E-Mail: Ulf.Gerdtham@nek.lu.se; 4 Division of Public Health Epidemiology, Department of Public Health Sciences, Karolinska Institutet, Stockholm, Sweden; E-Mail: Pia.johansson@ki.se

**Keywords:** lifestyle interventions, economic evaluation, Markov model, long-term effectiveness, primary prevention, secondary prevention, diabetes, cardiovascular disease, cost-effectiveness

## Abstract

Lifestyle interventions (*i.e.*, diet and/or physical activity) are effective in delaying or preventing the onset of diabetes and cardiovascular disease. However, policymakers must know the cost-effectiveness of such interventions before implementing them at the large-scale population level. This review discusses various issues (e.g., characteristics, modeling, and long-term effectiveness) in the economic evaluation of lifestyle interventions for the primary and secondary prevention of diabetes and cardiovascular disease. The diverse nature of lifestyle interventions, *i.e.*, type of intervention, means of provision, target groups, setting, and methodology, are the main obstacles to comparing evaluation results. However, most lifestyle interventions are among the intervention options usually regarded as cost-effective. Diabetes prevention programs, such as interventions starting with targeted or universal screening, childhood obesity prevention, and community-based interventions, have reported favorable cost-effectiveness ratios.

## 1. Introduction

Cardiovascular disease (CVD) and diabetes are the leading causes of death worldwide. An estimated 17.1 million people died from CVD in 2004, representing 29% of all global deaths. Diabetes causes approximately 5% of all deaths globally each year and its incidence is predicted to increase by over 50% in the next 10 years, according to the World Health Organization (WHO) [1]. People with diabetes develop CVD at an earlier age and are two to four times more likely to suffer strokes than healthy subjects, and approximately 73% of adults with diabetes are considered pre-hypertensive. These diseases also impose a substantial economic burden on individuals, families, and nations. Healthcare expenditures for diabetes are expected to account for 11.6% of total healthcare spending in the world in 2010 [2]. Besides excess healthcare expenditures, diabetes and CVD also impose costs in terms of lost productivity and foregone economic growth due to lost work days, lower work productivity, mortality, and permanent disability [3].

Lifestyle interventions, *i.e.*, changed dietary habits, increased physical activity, maintaining or reducing body weight, and smoking cessation, are effective in preventing CVD and diabetes. In recent decades, numerous studies have focused on preventing type 2 diabetes (T2DM) via lifestyle intervention. The Malmö feasibility study was the first [4], followed by other controlled trials, such as the Da Qing study in China [5], the Diabetes Prevention Program (DPP) in the USA [6], and the Diabetes Prevention Study (DPS) in Finland [7]. These trials have had a significant impact on public health policy, providing evidence of lifestyle interventions as preventive factors, and have been followed by similar studies in other countries, for example, India [8], Japan [9], and The Netherlands [10]. Reviews of lifestyle interventions have also indicated that diet and/or physical activity are effective in reducing CVD risk in primary care [11–14].

As evidence supports the role of diet and/or physical activity in preventing T2DM and CVD, preventive strategies should aim to reduce population-wide risk. Such risk reduction interventions, even if modest, could cumulatively yield substantial benefits. Given the considerable cost of such interventions, public health interventions are increasingly subject to economic evaluation [15–17]. Economic evaluations comprise the comparative analysis of two or more healthcare interventions in terms of their costs and consequences. The results of such evaluations help public health policymakers make informed decisions, ensuring that limited resources are allocated as efficiently as possible to improve overall population health while avoiding allocating resources to interventions with comparatively low cost-effectiveness [18,19]. The number of economic evaluations of diet and/or physical activity interventions focusing on T2DM and CVD is also increasing. There is one review of the cost-effectiveness of physical activity interventions, but not specifically regarding CVD or T2DM [20], one of dietary intervention to prevent CVD [21], and another of the cost-effectiveness of physical activity in treating disease [22]. There is also a review of economic evaluations of T2DM prevention [23], which updated three previous reviews. However, to our knowledge, no review has considered the health economic evidence regarding lifestyle interventions to prevent CVD and T2DM simultaneously, although these diseases share lifestyle risk factors (sometimes referred to as the metabolic syndrome [24,25]).

Modeling has become a crucial component of economic evaluations. Computer simulation models are usually a series of mathematical equations combined in a structural framework to allow the projection of short-term data from clinical trials to long-term health outcomes and costs [18]. Modeling is particularly relevant in the case of T2DM and CVD, since morbidity and mortality stem from chronic complications. Several models of diabetes and related complications and of CVDs have recently been developed [26,27]; for example, in the fourth Mount Hood Challenge, seven models of diabetes were analyzed [28]. There have been previous reviews of diabetes models [27,29], one of which concluded that models vary significantly in whether diabetes complications (micro *vs.* macro complications) are covered, and less in the detail of such coverage [29]. Several recent studies have incorporated the latest epidemiological data, enabling advanced modeling of diabetes and related complications.

Models incorporate the short-term outcome of an intervention and project its lifetime effects. Since lifestyle interventions aim to change subject behavior, the beneficial habits are supposed to continue after the interventions have ended. For example, in a followup study of DPS, the intervention group maintained the beneficial lifestyle changes with the relative risk reduction of 36% after the three-year followup of a 4-year intervention period [30]. Unlike DPS, the 10-year followup of DPP demonstrated that diabetes incidence was the same in the lifestyle and control groups (5.9 *vs.* 5.6), but that the cumulative incidence was lower in the lifestyle group, leading the authors to conclude that diabetes can be prevented or delayed for at least for 10 years by means of lifestyle intervention [31]. The 20-year followup study of Da Qing demonstrated that lifestyle intervention still had positive effects on the incidence of T2DM in the intervention group [32], but had no significant effect on CVD events, CVD mortality, or all-cause mortality relative to the control group. The DPS followup study also demonstrated that the effect of lifestyle intervention on 10-year CVD mortality was same in both control and intervention groups, unlike the Malmö preventive trial in which, after 12 years of followup, total mortality was lower for lifestyle participants [33]. Although there are unresolved issues concerning the long-term effectiveness of lifestyle interventions, economic evaluation of lifestyle interventions requires empirical evidence or logical assumptions to model probable future health outcomes.

This review critically appraises the literature, particularly seeking to answer the following questions:

1. How have economic evaluation of lifestyle interventions (*i.e.*, diet and/or physical activity) been implemented in preventing T2DM and CVD?
2. What models have been used in conducting these evaluations?
3. What assumptions have been made regarding the long-term effectiveness of interventions when modeling beyond the intervention period?

## 2. Methods

### 2.1. Search Process

We searched databases containing only economic studies, such as the British National Health Service Economic Evaluation Database (NHS EED) (available at http://www.crd.york.ac.uk/crdweb/), and cross-checked against the CEA registry (available at https://research.tufts-nemc.org/cear/search/search.aspx), as recommended by Pignone *et al.* [34] and done by others [35,36]. NHS EED contains articles from four major databases, *i.e.*, Current Contents–Clinical Medicine, MEDLINE, CINHAL, and EMBASE, starting from 1995. This database also includes studies from PsychLit, Biomed Central, paper-based journals, and other gray literatures. Using the search terms *Lifestyle*, *Diet*, and *Physical activity*, 115 lifestyle, 186 diet, and 146 physical activity articles were retrieved. All articles were exported to EndNote for review.

### 2.2. Inclusion and Exclusion Criteria

The first and last authors independently reviewed the article abstracts. The article search was limited to the 1995–2008 period. Duplicate articles were removed from EndNote, since some articles contained the same keywords. At this point, the CEA database was cross-checked for additional articles. The criteria for article selection were full economic evaluation, *i.e.*, cost-consequence analysis (CCA), cost-effectiveness analysis (CEA), cost-utility analysis (CUA), and cost-benefit analysis (CBA). Studies involving partial economic evaluation, cost of illness, or literature review were excluded. Only articles published in English were included. Studies unrelated to CVD or T2DM (e.g., studies of cancer or osteoporosis) were also excluded.

**Figure 1.**
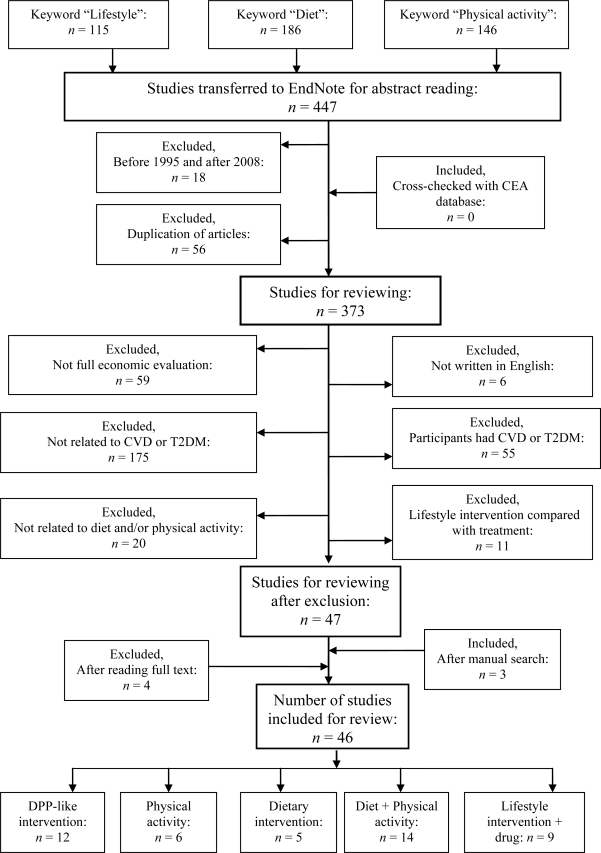
Flow chart for study selection for the review starting with the NHS EED database.

Mainly primary prevention, *i.e.*, participants were healthy at time of intervention, and secondary prevention, *i.e.*, participants were at high risk of developing diseases such as obesity or had impaired glucose tolerance (IGT)/impaired fasting glycemia (IFG), were included. Studies were excluded if participants had CVD or T2DM before intervention initiation. Studies were excluded in which lifestyle interventions (e.g., smoking cessation or reduced alcohol consumption) did not include dietary modification and/or physical activity. Pharmacotherapy was included if any lifestyle intervention was combined with drug treatment, or if it was a comparator for the analysis. Studies were excluded in which lifestyle interventions were compared with treatments such as gastric bypass surgery, therapeutic nutrition, and enteral nutrition. After fulfilling all criteria, 47 articles were selected for review; four articles were excluded after reading the full text, since the study participants had preexisting diabetes or CVD. Furthermore, the reference lists of articles were manually searched to find relevant articles, which added three articles. The review finally included a total of 46 articles. The search and article selection procedures are presented in Figure 1.

## 3. Results

The reviewed interventions vary from simple provision of information about behavioral changes to active participation and screening for diabetes or CVD, which might involve universal screening or targeted screening of high-risk groups. The comparator of the studies also varies, being placebo care, standard care, or lifestyle intervention alone, especially when pharmacological interventions are evaluated. The target groups range from school-aged children to subjects over 65 years old. The risk factors vary, the populations ranging from generally healthy to high-risk (*i.e.*, overweight, obese, and IGT/IFG positive), sometimes being gender specific (*i.e.*, five articles examined female participants and one examined males). The intervention settings are also diverse: some articles analyze new hypertension guidelines or national policies for countering overweight, others evaluate community or primary care settings, and three studies are school based. The evaluation countries are mainly developed ones, primarily the USA, followed by the UK, with only one study (the Indian Diabetes Prevention Programme—IDPP) from India. Only one study is a CBA; three are CCAs and the rest are either CEAs or CUAs. In the CUA, effectiveness is measured as quality-adjusted life years (QALYs), whereas in the CEAs the measure of effectiveness varies considerably, for example, being life years gained (LYG), incidence of T2DM prevented or delayed, percentage point decrease in 10-year CVD risk, or number needed to treat to prevent one case of diabetes. The effectiveness data are derived from single randomized controlled trials or from literature reviews of trials from the country of a particular intervention study, if available, and otherwise from other countries. Results are sometimes presented as the incremental cost-effectiveness ratio (ICER), *i.e.*, the ratio of the differences in costs between two alternatives to the differences in effectiveness between the same two alternatives (Tables 1–5 present only the results of lifestyle interventions). The discount rate ranges from 3% to 6%. Most studies use the same discount rate for costs and effects, although different rates are used, for example, in all studies from the Netherlands, where 4% is used for costs and 1.5% for effects. Sensitivity analyses are univariate, multivariate, or probabilistic. Of the 46 studies, 31 include decision analytic models (DAMs), such as decision trees, Markov models, and an Archimedes model.

Methodological variation affecting how results are derived makes intra- or inter-group comparison between the studies difficult. However, concentrating only on the monetary figures in the results and agreeing on what is considered cost-effective (50,000 US$/QALY, 20,000–30,000 £/QALY or 50,000 AU$/QALY), most lifestyle interventions are deemed cost-effective.

### 3.1. General Characteristics of the Studies

The papers are divided into five groups: (1) DPP-like lifestyle interventions, (2) physical activity interventions, (3) dietary interventions, (4) diet + physical activity interventions, and (5) drug treatment combined with any of the preceding interventions (Sections 3.1.1.–3.1.5.; Tables 1–5).

#### 3.1.1. DPP-Like Lifestyle Interventions

Economic evaluations of DPP-like lifestyle interventions have used different methodologies and produced inconsistent results. The first economic evaluation of DPP, which compared lifestyle interventions and pharmacotherapy (metformin) with placebo care, was performed by the DPP Research Group in 2003 [37]; it covered only the three-year intervention period without any DAM and from a societal perspective. As DPP intervention costs were very high, it was proposed that the intervention be offered on a group basis (10 people per group) instead of face to face; it was assumed that the effectiveness would be similar, so that the costs of lifestyle intervention would be reduced. As a three-year time horizon overestimates the treatment costs and underestimates the benefits of lifestyle intervention and metformin, the same research group later extrapolated the trial data into lifetime costs and benefits using a Markov model [38]. The progression of IGT to clinical onset of diabetes and from diabetes-related complications to death was assessed.

A later study [39] used the DPP data for five countries, *i.e.*, Australia, France, Germany, Switzerland, and the UK, applying a simple three-state Markov model (*i.e.*, alive with IGT, alive with T2DM, and deceased) over a lifetime horizon. Another study analyzed the DPP results from a different perspective [40], *i.e.*, whether investment in a DPP intervention program by a health insurer would cut costs. The model was same as that of the DPP Research Group but used for two time periods. The conclusion was that, if the insurer and Medicare shared the DPP intervention costs (24% borne by the insurer), the insurer would recover the investment via avoided future medical care costs.

Two studies examined [41,42] screening for diabetes among overweight and obese people followed by DPP intervention, while Icks *et al.* [42] studied the cost-effectiveness of DPP in a real-world setting, *i.e.*, when acceptance of and adherence to the intervention is low and the dropout rate is high. In addition to metformin, another drug, acarbose, was examined in Caro *et al.* [43] using a four-state Markov model of a Canadian population over ten years; this study estimated that lifestyle modification would prevent 117 cases of diabetes, while metformin and acarbose would prevent 52 and 74 new cases of diabetes, respectively. The lifetime cost and effectiveness of DPP was estimated by Eddy *et al.* [44] using an Archimedes model, which contains infinite health states. This is the only study estimating that DPP-like lifestyle intervention has a mere 0.1% chance of costing under 50,000 US$/QALY.

DPP or DPP-like interventions have been studied in other countries as well. IDPP was performed by Ramachandran *et al.* [45]; although the analysis examined only the trial period (three years), the cost-effectiveness ratio was much lower than for DPP. Galani *et al.* reported two studies [46,47] on lifestyle interventions for overweight and obese Swiss population groups, with assumed effectiveness taken from DPS. A seven-state Markov model over a lifetime horizon estimated that lifestyle intervention could be cost-effective depending on sex, age group, and threshold values. DPS effectiveness was applied to a Swedish population [48] using a Markov model, which included the cost of added life years.

Methodological disagreement is the main issue in DPP-like studies. The results of DPP interventions are reported as 8,800 US$/QALY or 62,600 US$/QALY depending on whether a Markov [38] or Archimedes model [44] is applied. If 50,000 US$/QALY is considered a cutoff value for cost-effectiveness, the same trial is cost-effective with one method but not the other. The disagreement stems from different model assumptions on the rates of progression to diabetes and complications [49,50]. Both authors provide arguments and counterarguments defending their assumptions [51,52].

Despite the disagreement, it was predicted that DPP-like lifestyle intervention would delay the onset of diabetes and lead to fewer complications, longer lives, and improved quality of life [50]. All the DPP/DPS/IDPP-like lifestyle interventions were cost-effective, except that reported by Eddy *et al.* [44], even if the intervention started with costly screening [41,42]. IDPP was much more cost-effective than similar programs in high-income countries, because the intervention cost was much lower even though staff went to participant homes to provide the intervention [45]. The cost-effectiveness ratio was in the cost-effective range when DPS was applied to a Swedish population [48], even though unrelated costs for added years of life were included, which is debatable [53].

#### 3.1.2. Physical Activity Interventions

Several methods have been used to determine the physical activity level of participants in the economic evaluation of physical activity studies. One study used metabolic equivalent (MET) minutes per week to classify participants as inactive, irregularly active, meets guidelines, and highly active [54]. Some studies used duration and intensity of physical activity, *i.e.*, ≥2.5 hours of moderate intensity or vigorous exercise per week as marking a physically active person [55], while other studies used the self-administered physical activity questionnaire (PAQ) [56,57]. There were only two Markov models [54,55] of physical activity: the CDC MOVE Markov model was based on five diseases (*i.e.*, coronary heart disease, ischemic stroke, T2DM, breast cancer, and colon cancer), which led to underestimated cost-effectiveness, as physical activity also affects other diseases such as depression or anxiety [58].

Indirectly measuring the level of physical activity (e.g., via PAQs) raises validity concerns [59]. The selection of participants for physical activity interventions is also a major concern, *i.e.*, whether the control group also participates in the program or only includes motivated people; for example, one study [56] excluded highly active participants. Participation rate and adherence to physical activity are other important issues meriting attention when modeling the long-term benefits of physical activity.

Surprisingly, the ICER of physical activity interventions is much lower in Australia [55] and New Zealand than in the USA [54] or UK [56]. This could be because Roux *et al.* [54] analyzed the physical activity promotional intervention using a model containing considerably more health states and because the participants were older in the Munro *et al.* [56] study; however, all ICERs are in the cost-effective range.

#### 3.1.3. Dietary Interventions

An economic evaluation of ten different nutritional interventions for the Australian population was performed by Dalziel and Segal [60]. Four Markov models were developed to analyze nutritional interventions. Reduction of dietary sodium intake to reduce hypertension, eating five servings of fruit and vegetables per day, and adhering to a “Mediterranean-style” diet seemed to be cost-effective. The effect of the Mediterranean diet was estimated to reduce the 10-year CVD risk based on the Framingham risk equation. The cost-effectiveness of grains fortified with folic acid as well as vitamin supplementation with folic acid and cyanocobalamin (vitamin B12) to prevent coronary heart disease (CHD) was analyzed in one study [61] of the entire US population via the CHD Policy Model. Nutritional education was compared in Cox *et al.* [62], one group receiving traditional face-to-face advice, while another received self-administered video lessons. The effectiveness of the intervention was analyzed using a behavior questionnaire on changes in 10 dietary factors.

There are few economic evaluations of dietary interventions and the quality of studies is often limited, since some important economic evaluation parameters (*i.e.*, age, risk factors, sensitivity analysis, and price year) are not clearly specified. The use of self-administered questionnaires to measure food intake raises validity issues.

Dietary interventions are very diverse, and probably not very comparable. Moreover, varying study quality makes it difficult to compare the results of the dietary interventions, but fruit and vegetable intake has the lowest cost-effectiveness ratio (46 AU$/QALY) [60] and similar results are obtained from universal fortification of grains with folic acid.

#### 3.1.4. Diet + Physical Activity Interventions

Three articles presented economic evaluations of a national policy or action plan [63–65]. The new Finnish hypertension care guidelines were compared with previous ones [64], which did not incorporate lifestyle intervention; the new guidelines were estimated to save an additional 49,000 life years. The Dutch national action plan for counteracting obesity included a community-based intervention in which 90% of the population was screened and received lifestyle advice; an intensive lifestyle program was applied to 10% of the overweight adults in a healthcare setting. The combined program was evaluated using the RIVM chronic disease model, finding that it would save 110,000 life years over 20 years [63]. Using a diabetes model [65], it was estimated that diabetes care and prevention strategies applied to the entire Australian population aged 45–74 years would prevent 53,000 new cases of diabetes over 10 years.

Three articles studied childhood obesity prevention programs [66–68]. The “traffic light diet,” physical activity reinforcement, self monitoring, and stimulus control were used as interventions in one study; their effectiveness was measured as a reduction in standardized body mass index (Z-BMI) and proportion of overweight children [67]. Another study [68] examined Planet Health, a school-based obesity prevention program focused on reducing television viewing, reducing consumption of high-fat foods, increasing fruit and vegetable consumption, and increasing moderate and vigorous physical activity. The randomized controlled trial included male and female subjects, but there were significant reductions in obesity only among female students after two school years. This intervention effect was then extrapolated up to age 64 years using a decision model measuring health benefits as QALYs. The same method was used in another study [66] of a different school-based obesity prevention program (CATCH) including male and female participants from grades three (age 8) to five (age 11). CATCH appears to be more cost-effective than the Planet Health program.

Two studies have focused solely on reduction of CVD incidence using the Framingham risk equation [69,70]. One study compared lifestyle interventions, including video and video + self-help guidelines, with routine care for Australian participants at risk of CVD [70]. In another study, community-based education to change dietary habits to reduce total cholesterol level was estimated to prevent approximately 4.5 cases of CVD every year, with the highest benefit in the 55–64-year age group, in which it would prevent 8–9 cases [69] in a population of 5,500. The WISEWOMEN project, which aimed to reduce CVD risk in older uninsured and undernourished women by means of either CVD screening + enhanced lifestyle intervention or CVD screening + minimal intervention, was evaluated in two studies [71,72]. The earlier study [72] measured effectiveness as the percentage reduction of the 10-year probability of having CVD, while the later study [71] measured it as LYG as well, resulting in a cost per LYG of US$ 4400. The effect of a single randomized controlled trial intervention of diet, exercise, and diet + exercise extrapolated on a cohort of 60-year-old healthy subjects without CVD was evaluated in one study [73]. The Markov model included 10 health states but not diabetes. The cost-effectiveness of interactive group sessions advising on nutrition and physical activity for cohabiting Australian couples was studied [74] on an intention-to-treat basis with outcomes measured on 14 variables (e.g., energy intake, fat intake, fiber intake, sodium, fruit and vegetable intakes, BMI, physical activity level, cholesterol profiles, and blood pressure). Two types of lifestyle intervention were evaluated by Jacobs *et al.* [75]—a community-based intervention for the general population covering many people at a relatively low cost, and a healthcare-based intervention for high-risk people covering fewer patients at a higher cost—using the RIVM chronic disease model.

Many studies lack detailed information about certain important economic evaluation parameters and on how data have been derived, for example, via meta analysis, systematic review, selective studies, or expert opinion. These parameters include cost (e.g., unit cost, total cost, intervention cost, disease cost, and productivity cost) [65,72,74], utility [63], and disability weight [65]. In childhood obesity studies [66,68], the explicit assumption requires evidence, which is missing, from epidemiological studies linking childhood overweight to adult overweight and to weight regain after two-year interventions. The same issue applies in CVD cases: a time lag after termination of lifestyle intervention in improvements of CVD risk factors. Lindholm *et al.* [69] considered a five-year lag, whereas

Dzator *et al.* [74] considered no time lag. The transferability of one country’s clinical trial data to another [65] also requires adjustment. For community-based interventions [63,69,75], special attention is required to consider the spillover effect of lifestyle interventions and the efficacy of interventions in various socioeconomic groups, *i.e.*, the equity concern is missing from the studies. However, the main question concerns the applicability of intervention effectiveness data from clinical settings to real-world settings, particularly when national action plans or policies are being evaluated [63–65].

Interventions starting from childhood have a low cost-effectiveness ratio (900 US$/QALY) [66]. The community-based approach is also attractive, as seen in the Dutch [63,75] and Swedish studies [69]. The advantage of community-based lifestyle prevention programs is that the health gains achieved through population-based approaches often exceed those achievable by targeting specific groups in clinical or subclinical settings.

#### 3.1.5. Combined Drug and Lifestyle Interventions

Three articles [76–78] concerning the drug sibutramine were studied by the same research group using the same model but in different country settings, *i.e.*, Finland, Germany, the USA, the UK, and Switzerland. Lifestyle intervention was included along with the drug to treat overweight or obese people. The total number of fatal and non-fatal CHD events avoided in five years of analysis was estimated as 3.49 in Finland, 4.18 in Germany, 4.49 in Switzerland, and 1.96 in the UK per 1,000 people, while the average number of diabetes cases avoided was 3.0. In an Italian obese population, another drug, orlistat, was studied [79]. The same drug combined with a low-calorie diet for obese patients was also assessed for the whole Dutch population, estimated over a lifetime horizon using the RIVM chronic disease model [80]; the combined therapy was estimated to produce an additional 34,000 life years.

A weight-reduction drug, rimonabant, was compared with lifestyle intervention [81] using a decision tree with five arms, *i.e.*, treatment options. Two years’ treatment with rimonabant combined with lifestyle intervention produced the most cost-effective option. Employing a Markov model, Roux *et al.* [82] compared four weight-loss strategies in overweight and obese women. The strategies were diet only, diet + pharmacotherapy (orlistat), diet + exercise, and diet + exercise combined with behavior therapy. The weight-loss intervention consisted of six months of intervention followed by a six-month maintenance program. Diet + exercise combined with behavior modification was the best strategy.

When statin drugs and cholesterol-lowering diets were compared with no interventions for patients with a high cholesterol level using the CHD Policy Model over a 30-year period, both interventions produced much higher cost/QALY in the primary prevention [83]. This may be because statins were expensive at the time (1997), before they were available as generics. In another study [84], four strategies for preventing or delaying diabetes, *i.e.*, screening for early detection, screening + lifestyle intervention, screening + lifestyle + pharmacological intervention, and no screening, were compared with a hybrid model using a three-arm decision tree and seven-state Markov model. Screening followed by lifestyle intervention was the most cost-effective strategy.

All pharmacological studies included a short-term trial period (six months to one year) extrapolated to a five- or ten-year time horizon, except one that employed a lifetime horizon [80]. One issue in pharmacological interventions was weight regain after intervention end; the National Institute of Clinical Excellence (NICE) recommendation [85] on assumed uniform weight regain over a three-year period had not been taken into consideration. Furthermore, though all drugs had some side effects, the related loss of quality of life was not considered. We agree with previous review findings that the main sources of uncertainty in pharmacological interventions are weight-loss sustainability, utility gains associated with weight loss, extrapolation of long-term benefits from short-term trials, dropout rate, side effects, and bias towards the funding authority [86].

Surprisingly, all effectiveness data for drug interventions are based on literature reviews, except data from the study [79] reporting the highest ICER (75,300 €/QALY). When lifestyle interventions are compared with pharmacotherapy, lifestyle interventions are more effective, *i.e.*, in survival years, disease-free time, and quality-adjusted life expectancy [82], indicating that lifestyle interventions are better options for preventing lifestyle diseases.

#### 3.2. Decision Analytic Models (DAM)

Of 46 studies, 31 employed DAM: six used decision trees, 20 used Markov models, and one used an Archimedes model (Table 6). One article used both a Markov model and a decision tree [84], one used four Markov models [60], one used an Archimedes model [44], and two used a life table approach. A decision tree is a simple visual representation of possible options and their consequences. Decision trees start with the options, each of which branches out to explore all potential health outcomes and their respective probabilities and costs. In Markov models, participants move from defined health states (Markov states) in discrete time periods (Markov cycles). Each health state incurs particular costs and health consequences [18]. It is common to use a previously developed model; all the

Dutch [63,75,80] studies used the RIVM chronic disease model adapted to the study objectives, while the CHD Policy Model [61,83] and the CDC diabetes model [38,40,41] have been used in many studies.

Philips *et al.* [92] emphasized several issues for good practice in modeling: model structure; data employed, *i.e.*, inputs (costs) and outcomes (health benefits); and model consistency or validity. For Markov models, the structure generally concerns the health states included, as inclusion of costly diseases (e.g., stroke due to diabetes in diabetes progression models) might overestimate the long-term results. Another example is that the RIVM chronic disease model [63] included certain types of cancer absent from another model [46], although both models were developed for overweight and obesity.

The effectiveness data were taken from a single trial or literature review. For most models, the model population was a hypothetical cohort but, when the effectiveness of a trial was transferred to different country settings, the model population was often matched with the study population; for example, the survey population from the US National Health and Nutrition Survey [41] and the participants in the German KORA study were matched with the DPP participants [42], and similarly in three other studies [39,44,60].

Two types of cost data were included in the models: cost of intervention and cost of disease avoided. Some studies estimated the intervention cost from a clinical trial [91], some calculated the cost retrospectively [71], and some based the cost on national administrative databases [65], expert opinion [73], or even modeler opinion. The avoided disease costs were country specific if available; otherwise, the applicable data were taken from other countries. The model outcomes were QALYs, DALYs, LYG, or other measures of health. Different countries were found to have used different instruments when estimating QALYs: QWB-SA was used for the DPP models, European studies frequently used EQ-5D, while Finnish and Australian studies preferred their own instruments (*i.e.*, 15D and Aqol). Models are subjected to internal validation (*i.e.,* comparing model output with the data used in building model), external validation (*i.e.*, checking whether the model output is consistent with the disease outcome and epidemiological data), and between-model validation (*i.e.*, comparing the estimated intervention outcome with the outcomes of other models based on similar assumptions and addressing similar diseases) [92,93], as model quality depends largely on input data quality [94]. No studies provided any details on all the three types of validation. However, in modeling, it is recommended that a technical report [19], *i.e.*, a detailed description of all assumptions and parameter values used to construct the model, be provided. Not all model studies mentioned technical reports, though some provided supplementary materials.

### 3.3. Long-Term Effectiveness of Lifestyle Intervention

An important expectation in a lifestyle intervention is long-term adherence to the changed behavior, either the change in dietary habit or the increase in physical activity. A key issue in economic evaluations of such interventions is to link short-term evidence from clinical trials or epidemiological data to the long-term benefits of changed behavior. When considering long-term effectiveness, researchers often analyze different scenarios with optimistic or pessimistic assumptions and then assess the uncertainty in sensitivity analyses. However, there is some indecision concerning the optimistic and pessimistic assumptions or the best- and worst-case scenarios. The base case assumptions used in the lifestyle interventions, alternatives used for sensitivity analyses and the changes in baseline results of the sensitivity analysis are highlighted in Table 7.

The assumption about whether the intervention effect is maintained after the intervention (or trial) dramatically affects the cost-effectiveness ratio or result. We believe it is pessimistic to assume that the effectiveness will persist only as long as the intervention period, as has been done in several studies [39,63,65,70,75], and too optimistic to assume that the effectiveness will persist until death, as is done elsewhere [71,73]. For example, one study assuming that the effectiveness would persist only for the intervention period (pessimistic assumption) reported a result of 152,000 AU$/QALY; however, if the effectiveness had been assumed to persist one additional year, the result would be 6,600 AU$/QALY [70]. On the other hand, if intervention effectiveness is assumed to be one year (pessimistic assumption), instead of lifelong the cost-effectiveness ratio would be 10 times higher than the base case (lifelong effectiveness) result [71]. In the case of DPP, some researchers assumed that the intervention and its effectiveness would persist until the participants developed diabetes or died— an optimistic assumption [38,40,41,44]. On the other hand, other researchers assumed that the effectiveness would decline by 20% each year [41] and 50% over the total period [38,40]. Reducing the effectiveness by 20% resulted in 1.5 times [41] and almost seven times [38] higher total cost-effectiveness ratios than in the base case analysis. In the DPP trial, the lifestyle intervention was 58% effective, which Caro *et al.* [43] used for the base case analysis with 30% as the worst- and 70% as the best-case value. With 70% effectiveness, the result was dominant, *i.e.*, the intervention is more effective and less costly than the alternative. Roux *et al.* [82] assumed in the base case analysis that only 20% of participants would maintain the changed behavior in the long term, with optimistic and pessimistic assumptions, *i.e.*, over 40% and under 10%, respectively, which resulted in estimates four times higher or half the base case cost-effectiveness ratio. Van Baal *et al.* [80] assumed that 23% of the intervention weight loss would persist in the long run as the pessimistic assumption, with optimistic values of 50% or 100% maintained weight loss. With 100% maintained weight loss, the cost-effectiveness ratio was almost three times lower than the base case value.

When long-term effectiveness issues are addressed using univariate sensitivity analysis, the changes in results are obvious. However, when probabilistic sensitivity analysis is performed, the changes in results due to effectiveness uncertainty are not clearly distinguished, as seen in two studies by the same author [46,47]. The assumptions in these studies were that weight loss (the intervention effect) would persist up to six years and the regain process would take four more years. After 10 years, the weight of the participants would be same as at the intervention start.

For the pharmacological weight reduction, the weight regain process was often assumed to be completed within five years of a one-year intervention [76–78], and a confidence interval (CI) was used in the sensitivity analysis. Assuming one year of sustained weight reduction [81] made the cost-effectiveness ratio unfavorable. Nevertheless, if the weight loss persisted for three years, the value was in the cost-effective range (under US$ 50,000); using the probabilistic sensitivity analysis, the authors demonstrated that at US$ 50,000 willingness to pay, the intervention had 40% chance of being cost-effective.

## 4. Discussion

It is difficult to compare the results of one cost-effectiveness analysis with another, because of differences in methodology, types of costs included, outcomes, and population groups and related baseline risk. There may also be differences in, for example, healthcare systems, incentives to healthcare professionals and institutions, clinical practices, population values, availability and accessibility of technologies, and currency purchasing power.

Establishing that an intervention is cost-effective is still problematic, since the threshold for cost-effectiveness, *i.e.*, decision maker willingness to pay, is controversial. NICE in the UK uses a cost-effectiveness threshold range of £20,000 to £30,000 per QALY gained [95,96]. In contrast, there are no official guidelines for the USA and Australia, though US researchers frequently employ 50,000 US$/QALY [97], while Australian researchers use 50,000 AU$/DALY [98] as thresholds. WHO has recommended that interventions be considered cost-effective if costs per DALY are 1–3 times GDP per capita [99]. Some argue that cost-effectiveness thresholds may be too high [100,101], others argue that they are too low [102,103], while still other claim the well-accepted US$ 50,000 threshold is misused [104]. Moreover, one review of cost-effectiveness analyses suggested that published studies tended to report results below US$ 50,000 per QALY [105].

Some recent DAM guidelines emphasize that models should be kept as simple as possible, providing they capture all essential parts of the disease processes, including effects of health technologies, to help policymakers make informed decisions [93,106–108]. Certain standard criteria should be considered when developing a model, in what is often referred to as validating [109,110] and calibrating [94,111] a model. It is not always possible to apply all recommendations in one model, so researchers often make tradeoffs between model accuracy and transparency. Transparency refers to the understandability of the logical arguments of a model, to enable it to be reproduced; accuracy refers to a model’s ability to capture real-life situations [112]. Balance between accuracy and transparency is difficult to obtain in a model: as a model is made more accurate, its complexity increases, which in turn reduces its understandability to decision makers. Accordingly, some researchers emphasize model transparency [93], whereas others argue that accuracy should be paramount [112].

When an intervention leads to significant health benefits in comparison with the comparator, the ICER is supposed to be low. For example, when a physical activity prescription was effective in a target population in New Zealand, the ICER was very low [55]; however, when interventions had no significant impacts on target groups, as seen in two studies of video-based lessons [62,70], the interventions were less cost-effective. Another aspect is the analytical time horizon: if it is short, health benefits are limited, likely resulting in high ICERs. DPP provides a good example; the ICERs for the short-term, three-year trial period [37] are much higher than those for the long-term lifetime analysis [38]. The age of the target population might also affect ICERs, as young target groups might achieve greater health benefits than older groups. Interventions for school children are very beneficial [66], as are those starting at ages around 20 [54,75,76]. In contrast, interventions starting later in life, such as those examined by Lindgren *et al.* [48,73], who use 60 years as the starting age, result in much higher ICERs. ICERs are also affected by the risk level of the population. When the risk is high, as it is among the overweight or obese, the potential health gains from interventions are higher; this was illustrated nicely in two articles [46,47].

Naturally, intervention cost drastically affects cost-effectiveness, as is obvious in the DPP trial of costly face-to-face *vs.* cheaper group-based counseling. Another aspect is whether screening for high-risk individuals is included in the interventions. Icks *et al.* [42] reported that diabetes screening comprises 36% of the total intervention cost, which was one reason for the high ICER. The costs of developing a website [91] to motivate participants to increase physical activity can also make the cost-effectiveness ratio unattractive.

The cost-effectiveness ratio of our reviewed studies range from 46 AU$/QALY [55] for fruit and vegetable intake to as high as 143,000 US$/QALY for DPP lifestyle intervention [44]. Community-based interventions seem to have low cost-effectiveness ratios [63,69,75] ranging from 1,100 to 5,000 Euro/QALY. School-based interventions are also attractive, at 900 US$/QALY [66] and 4,305 US$/QALY [68], as are targeted screening followed by lifestyle interventions [41,42]. However, any targeted intervention could be made more favorable by dealing with the abovementioned issues (*i.e.*, risk level in target groups, intervention cost, intervention effectiveness, and starting intervention at young age) and adjusting the assumptions of the model parameters. So, cautious interpretation is required to generalize the results.

Icks *et al.* [42] argued that we lack information on the long-term effects of T2DM prevention interventions, and lack valid data regarding the natural course of T2DM from early onset to death. However, recent studies have examined the long-term effects of the DPP [31], Da Qing [32], and DPS trials [113]. This suggests that it is time to update models, so they are based on recent epidemiological data. Nevertheless, if long-term clinical effectiveness data are unavailable, the only way to explicitly explore the future effect of an intervention after its completion is by modeling; that modeling permits such exploration should be considered one of its major strengths, not a weakness [114]. The validity of long-term effectiveness assumptions would, however, benefit from some kind of consensus and harmonization, apart from the recommendation to perform relevant sensitivity analyses.

This review is limited in that it is not systematic and in that it omits studies not included in the NHS-EED database, such as monographs, some gray literatures, and book chapters. Smoking cessation is an important lifestyle intervention but, as the topic has been subject to extensive review [115,116], it is excluded here. Dieting is a popular lifestyle intervention, but we found no articles focusing specifically on dieting as a weight-loss intervention. However, one objective of DPP-like interventions was to reduce weight by 7% using both diet and physical activity interventions. Roux *et al.* have used diet as a sole intervention for weight loss in women [82], but three other weight-loss strategies were also addressed at the same time. Another study [73] used a sole dietary intervention, though the objective was not weight loss. The ICERs in the studies have not been converted to a common price year, since there is no fixed cost-effectiveness threshold, and the ICERs reported depend on the comparators, which vary widely in the studies reviewed. The actual incremental costs per health outcome reported are better regarded as indications of cost-effectiveness. Another important limitation is that none of the three available quality checklists [117–119] is used for assessing article quality, partly because the checklists were developed only recently [117,119], after several of the included studies. Furthermore, a recent study states that the quality appraisals depends on the researchers and not on the checklists [120], as ICERs are unlikely to be affected by a single factor but rather by a combination of several.

This is the first study, to our knowledge, to include dietary and physical activity lifestyle interventions that affect T2DM and/or CVDs, with a special focus on DPP-type interventions. A recent study by Anderson [121] questioned the use of systematic reviews of economic evaluations, partly because the interplay of 26 factors makes the results of cost-effectiveness analyses vary depending on setting and location [122]; as well, 14 factors had to be considered to ensure transferability of results from one country to another [123]. On the other hand, there are three good reasons to review economic evaluations: (1) to study the development of new decision models; (2) to identify the most relevant studies for a particular decision making context; and (3) to identify the “how and why” causality of interventions that are cost-effective in certain settings but not in others, including the principal economic tradeoffs in particular decision areas. This review identifies several new decision models, such as models of screening for diabetes and ensuing interventions [84], a model of multiple behavior modification [82], a model of cardio–metabolic disorders [46], and a model used for DPS [48] that differs from older models, such as the CHD Policy Model [61,83], the Johannesson model [70], and the CDC model [38,40–41]. The MOVE model [54] is a new updated physical activity model, differing from a previous model developed by NICE [124]. The models developed to study national plans of action/policy are new and advanced, and economic evaluations of national action plans may well constitute a new research area [63–65]. The economic tradeoff in intervention options is well demonstrated in DPP-like studies, in which intervention provision (individual *vs.* group counseling) and different country settings (USA *vs.* India) are key factors [37,45]. Tradeoffs have also been identified in Dutch studies in which community-based, high-cost/low-effectiveness intervention is compared with healthcare-based highly effective low-cost intervention [63,75]. The explanatory theory-building aspects of how and why an intervention works are hard to establish in public health interventions because of the complex and inherent interplay of several health determinants. Moreover, the reviewed studies were not informative enough in explaining how the intervention, cost, and health effect outcomes are affected by different configurations of input variables (e.g., patient characteristics and context) to build such theory [121]. However, it was found that using different modeling techniques with different assumptions leads to different results, as in the Archimedes model [38] and the Markov model [44] in the DPP trial.

## 5. Conclusions

We believe that demand for economic evaluations will continue, because of the need to assess the growing number of interventions available to prevent and treat diseases. Economic evaluations of public health programs are still comparatively new and might differ in some respects from conventional economic evaluations [36,125]. Further work is also needed to determine the cost-effectiveness of interventions in disadvantaged populations and to examine the related issue of equity.

Widespread implementation of lifestyle interventions in high-risk groups to prevent T2DM and CVD has no obvious drawbacks. Furthermore, as lifestyle interventions also reduce the risk of other chronic diseases, including certain forms of cancer, they have broader benefits for health. Unlike drug treatments, lifestyle interventions have few side effects. The use of DAMs in economic evaluations does not change the fact that cost-effectiveness analysis cannot incorporate all the values and criteria relevant to health policy decisions; it can, however, help to inform decisions in a direct manner.

Lifestyle interventions appear cost-effective in reducing the long-term risk of T2DM and CVD. It is even cost-effective to screen, either targeted or universally, for diabetes and CVD. Combined interventions, for example, diet and physical activity, are more beneficial than sole dietary or physical activity interventions. Interventions starting from school-aged children or focusing on the whole community are attractive in terms of cost-effectiveness.

## Figures and Tables

**Table 1.** General characteristics of articles on DPP, DPS, and IDPP.

**First author, year**	**Intervention**	**Comparator**	**Age, risk factor**	**Country, type of EE**	**Intervention period**	**Perspective**	**Effectiveness measure**	**Effectiveness source**	**Results and conclusion**	**Price year, discount rate**	**Sensitivity analysis**	**Model**
Ackermann ’06 [40]	DPP lifestyle intervention	Standard care	≥25 y, BMI ≥ 24, IGT	USA, CUA	3 years	Healthcare	QALY	Single study (DPP)	1,288 US$/QALY	2000, 3%	Univariate	DAM
Caro ’04 [43]	Acarbose, intensive lifestyle intervention, metformin	No intervention	40–70 y, BMI > 25, IGT	Canada, CEA	5 years	Healthcare	Preventing diabetes, LYG	DPP, DPS, and for acarbose STOP-NIDDM trial	ICER Lifestyle intervention 749 *vs.* no intervention, 7,252 *vs.* metformin, 9,988 *vs.* acarbose (CA$/LYG)	2000, 5%	Univariate	DAM
DPP RG ’03 [37]	DPP lifestyle intervention	Standard care	≥25 y, BMI ≥ 24, IGT	USA, CUA	3 years	Healthcare and societal	Per case of diabetes delayed/prevented, QALY	Single study (DPP)	51,600 US$/QALY societal perspective	2000, 3%	Univariate	No model
Eddy ’05 [44]	DPP lifestyle intervention, no intervention initially then dietary advice, no intervention initially then DPP, metformin.	No intervention	Adult, BMI > 24, fasting plasma glucose 5.27–6.93 mmol/L	USA, CUA	3 years	Healthcare and societal	QALY	DPP and literature review	143,000 US$/QALY healthcare and 62,600 US$/QALY societal perspective for DPP lifestyle intervention	2000, 3%	Univariate	Archimedes model
Galani ’07 [47]	Lifestyle intervention (DPS)	Standard care	≥25 y; overweight BMI 25–29.9, borderline BMI 30, moderate obese BMI > 30	Switzerland, CEA, CUA	3.2 years	Societal	LYG, QALY	Literature review	64 CHF/QALY for females and 354 CHF/QALY for males in borderline group	2006, 3%	Probabilistic	DAM
Galani ’08 [46]	Lifestyle intervention (DPS)	Standard care	≥25 y, overweight BMI 25–29.9, borderline BMI 30, moderate obese BMI > 30	Switzerland, CUA	3.2 years	Societal	QALY	Literature review	ICER 4,358 CHF/QALY (females) and 2,189 CHF/QALY (males), 30 years old and overweight	2006, 3%	Probabilistic	DAM
Herman ’05 [38]	DPP lifestyle intervention	Standard care	≥25 y, BMI ≥ 24, IGT	USA, CUA	3 years	Healthcare and societal	QALY	Literature review	1,100 US$/QALY healthcare and 8800 US$/QALY societal perspective	2000, 3%	Univariate, probabilistic	DAM
Hoerger ’07 [41]	Targeted screening (IGT & IFG positive) and either IGT or IFG positive + lifestyle	No screening	45–74 y, BMI ≥ 25	USA, CUA	until participants get diabetes	Healthcare	QALY	Literature review	8,181 US$/QALY for (IGT + IFG) and 9,511 US$/QALY for (IGT/IFG)	2001, 3%	Univariate	DAM
Icks ’07 [42]	Targeted screening + lifestyle, targeted screening + metformin	No intervention	60–74 y, BMI ≥ 24	Germany, CEA	3 years	Healthcare and societal	Incidence of T2DM avoided	DPP and literature review	4,664 Euro healthcare and 27,015 Euro societal perspective per case T2DM avoided by lifestyle intervention	2004, NP	Univariate, probabilistic	DAM
Lindgren ’07 [48]	Lifestyle intervention (DPS)	No intervention	60 y, BMI > 25, fasting glucose > 6.1 mmol/L	Sweden CUA	3 years	Societal	LYG	Single study (DPS)	ICER 127,065 societal and 98,725 healthcare perspective with declining effect and 141,555 societal and 11,642 healthcare with remaining effect (SEK/LYG)	2000, 3%	Univariate	DAM
Palmer ’04 [39]	Intensive lifestyle advice, standard lifestyle advice + metformin	Standard lifestyle advice	≥25 y, mean body weight 94.2, mean BMI 34	Australia, UK, France, Germany, Switzerland, CEA	3 years	Healthcare	LYG, years free of T2DM	DPP and literature review	Country specific; lifestyle and metformin were cost saving in all countries except UK	2002, 5% (UK 6% cost, 1.5% effect)	Univariate	DAM
Ramachandran ’07 [45]	Lifestyle intervention, metformin, lifestyle intervention + metformin	Standard lifestyle advice	35–55 y, reproducible IGT	India, CEA	3 years	Healthcare	Preventing one case of diabetes	Single study (IDPP)	Lifestyle intervention 1,052 US$, lifestyle + metformin 1,359 US$ per case of diabetes prevented	2006, NP	Univariate	No model

**Table 2.** General characteristics of articles on physical activity (PA).

**First author, year**	**Intervention**	**Comparator**	**Age, risk factor**	**Country, type of EE**	**Intervention period**	**Follow up**	**Perspective**	**Effectiveness measure**	**Effectiveness source**	**Results and conclusion**	**Price year, discount rate**	**Sensitivity analysis**	**Model**
Dalziel ’06 [55]	Prescription-based PA counseling by GP	Standard care	40–79 y, not active	New Zealand, CUA	3 weeks to 2 years	-	Healthcare	Number of participants became active, QALY	Single study (RCT)	ICER 2,053 NZ$/QALY	2001, 5%	Univariate, probabilistic	DAM
Munro ’04 [56]	Twice weekly physical exercise	No intervention	≥65 y, not active	UK, CUA	2 years	-	Healthcare	QALY	Single study	ICER 17,174 €/QALY	2003/2004, NP	Not clear	No model
Roux ’08 [54]	Promotion of PA	No intervention	25–64 y	USA, CUA,CEA	-	-	Societal	QALY	From 7 trials and the literature	ICER 14,286 to 68,557 US$/QALY	2003, 3%	Univariate, probabilistic	DAM
Sevick ’00 [87]	Lifestyle PA (behavioral skill training to increase PA)	Structured PA (prescription, supervised, centre based)	35–60 y, >140% ideal weight	USA, CCA	6 months	24 months	Healthcare	Several consequences for PA level and cardio-respiratory fitness	Single study	Lifestyle intervention is cost-effective	Not mentioned, 5%	Univariate	No model
Stevens ’98 [57]	Prescription-based PA	No prescription	45–74 y, Not active	UK, CEA, CCA	10 weeks	8 months	Healthcare	Moving a person from sedentary to physically active level	Single study (RCT)	2,500 £/person moving from inactive	Not mentioned, NP	Univariate	No Model
Sims ’04 [88]	Exercise counseling by GP	Standard care	20–75 y, not active	Australia, CEA	1 year	-	Healthcare	DALY saved and percentage of patients become active	Single study (RCT)	138 AU$/patients become active, 3,647 AU$/DALY	1996, NP	Univariate	No model

**Table 3.** General characteristics of articles on dietary interventions.

**First author, year**	**Intervention**	**Comparator**	**Age, risk factor**	**Country, type of EE**	**Intervention period**	**Perspective**	**Effectiveness measure**	**Effectiveness source**	**Results and conclusion**	**Price year, discount rate**	**Sensitivity analysis**	**Model**
Cox ’03 [62]	Face-to-face food behavior changing session	Self-administered video lesson	15–52 y, low income	USA, CEA	3 months	Not mentioned	A behavior checklist and intake of various nutrients	Single study	Video lesson was less costly 4,820 (US$) than face-to-face lesson 13,463 (US$)	Not mentioned	Not clear	No model
Dalziel ’07 [60]	10 nutritional interventions	Details of all comparators not provided	-	Australia, CEA, CUA	12 months	Societal	QALY	Literature review	Mediterranean diet 1,020, intensive lifestyle intervention 1,880, media campaign for 2 fruits & 5 vegetables 46, media campaign for fighting fit, fighting fat 5,600 (AU$/QALY)	2003, 5%	Univariate	DAM
Joffers ’07 [89]	Reduction in dietary sodium consumption	Standard care	-	Canada, CEA	1 year	Not mentioned	Decrease in hypertension prevalence, cost savings	Literature review	430 million CA$/year	Not clear, NP	NP	No model
Panagiotakos ’07 [90]	People having diet close to Mediterranean diet	People having traditional diet	Adults	Greece, CEA	-	Not mentioned	Time free of the development of CHD and life years lost	Single study (RCT)	ICER 50,989 Euro for additive healthcare cost due to non-Mediterranean diet for each year lost	Not mentioned	NP	No model
Tice ’01 [61]	Grain fortification with folic acid and also vitamin supplementation	No fortification	35–65 y	USA, CUA	-	Healthcare	Reduction in CHD events, medical cost savings and QALY saved	Literature review	For men ≥ 45 years, 300,000 QALYs and women >55 years, 140,000 QALYs will be saved in 10 years	1997, 3%	Multivariate	DAM

**Table 4.** General characteristics of articles on diet + physical activity.

**First author, year**	**Intervention**	**Comparator**	**Age, risk factor**	**Country, Type of EE**	**Intervention period**	**Perspective**	**Effectiveness measure**	**Effectiveness source**	**Results and conclusion**	**Price year, discount rate**	**Sensitivity analysis**	**Model**
Bemelmans ’08 [63]	Lifestyle intervention, community-based approach, combined intervention	No intervention	20–80 y, overweight for lifestyle	Netherlands, CEA, CUA	-	Healthcare	LYG and QALY	Two Dutch studies, QALY from literature	Lifestyle 7,400, Community-based approach 5,000, Combined program 5,700 (€/QALY)	2004, 4% to cost and 1.5% to effect	Univariate	DAM
Booth ’07 [64]	New antihypertensive, current care guidelines including lifestyle counseling	Previous guidelines	40–74 y	Finland, CEA, CUA	-	Healthcare	LYG	National Health Examination Survey	New guidelines saved 498 million Euro and 49,000 LYG	2001, 5%	Univariate	DAM
Brown ’07 [66]	Dietary habits and physical activity changes in school curriculum	No intervention	8–11 y, BMI ≥ 85th percentile	USA, CUA, CBA	3 years	Societal	QALY, net benefit	Single study	900 US$/QALY, Net benefit US$ 68,125	2004, 3%	Probabilistic	No model
Colagiuri ’08 [65]	Screening and preventing diabetes by means of lifestyle activities	No intervention	55–74 y and high risk 45– 54 y, obesity, hypertension, family history of diabetes	Australia, CUA	-	Not clear	DALY	Epidemiological data from Australia, DPP, DPS, UKPDS	50,000 AU$/DALY	2000, 3%	Univariate	DAM
Dzator ’04 [74]	Information given by mail, mail + active participation	No intervention	Cohabiting couples	Australia, CEA, CCA	4 months	-	Changes in 16 variables, e.g., consumption of fat, fiber, fruit, and vegetables; BMI, PA, physical fitness, LDL, BP	Single study (RCT)	445.30 AU$/participant per unit change of outcome variable	Not mentioned	Univariate	No model
Finkelstein ’02 [72]	CVD screening + enhanced lifestyle intervention	CVD screening + minimum lifestyle intervention	>50 y, low income	USA, CEA	1 year	Healthcare	Percentage point decrease in 10-year probability of CHD	Single study	637 US$/percentage point reduction in CHD risk via intensive lifestyle	Not mentioned, 3%,	NP	No model
Finkelstein ’06 [71]	Screening, intervention including nutrition, physical activity, smoking cessation	No intervention	40–64 y, low income, uninsured	USA, CEA	1 year	Healthcare	Percentage point decrease in 10-year probability of CHD and LYG	Single study	470 US$/percentage point reduction in CHD risk, 4400 US$/LYG	Not mentioned, 3%	Univariate	No model
Goldfield ’01 [67]	Family-based behavioral treatment in group + individual basis	Group treatment only	8–12 y, 20–100% overweight	USA, CEA	12 months	Healthcare	Percentage overweight change for children and parents, reduction in Z-BMI	Single study (RCT)	Group treatment is more cost- effective	Not mentioned	Not clear	No model
Jacobs ’07 [75]	Community intervention for total population, healthcare intervention for people at risk	No intervention	20–80 y, 30–70 y, obese for intensive lifestyle	Netherlands, CUA	5 years for community, 3 years for healthcare	Healthcare	QALY and number of participants need to treat to prevent one case of diabetes or CVD in 20 years	Literature review	3,100–3,900 €/QALY for community intervention and 3,900–5,500 €/QALY for healthcare intervention	2005, 4% to cost and 1.5% to effect	Univariate	DAM
Lindholm ’96 [69]	Screening + advice on lifestyle changes	No intervention	30–60 y, living in higher CVD mortality community	Sweden, CEA	6 years	Societal	Change in serum cholesterol level, blood pressure, LYG	Single study	1,100 to 4,050 £/LYG	1992, 5%	Univariate	No model
Lindgren ’03 [73]	Diet, exercise, diet + exercise	No intervention	60 y, No CHD	Sweden, CEA	6 months	Healthcare and societal	LYG	Single study	ICER 127,065 from societal and 98,725 from healthcare with declining effect and 141,555 from societal and 11,642 from healthcare with remaining effect (SEK/LYG) for diet	2000, 3%	Univariate	DAM
Mcconnon ’07 [91]	Use of website for changes in diet and physical activity	Routine information in primary care	>40 y, BMI > 31	UK, CUA	12 month	Not clear	Changes in weight and BMI, QALY	Single study (RCT)	ICER 39,248 £/QALY	Not mentioned	Probabilistic	No model
Salkeld ’97 [70]	A video-based lifestyle change program, a video + self-help program	Standard care	18–69 y, one or more CVD risk factor	Australia CEA, CUA	12 months	Societal	LYG, QALY	One Australian trial and literature review	ICER 152,128 AU$/QALY for males in video + self help	1994, 5%	Univariate	DAM
Wang ’03 [68]	Dietary habits and physical activity changes in school curriculum	No intervention	14 y, BMI ≥ 85th percentile	USA, CUA	2 years	Societal	QALY, adulthood overweight prevented	Single study (RCT) and others	4,305 US$/QALY	1996, 3%	Univariate multivariate	DAM

**Table 5.** General characteristics of articles on combined drug and lifestyle intervention.

**First author, year**	**Intervention**	**Comparator**	**Age, risk factor**	**Country, type of EE**	**Intervention period**	**Followup**	**Modeling**	**Perspective**	**Effectiveness measure**	**Effectiveness source**	**Results and conclusion**	**Price year, discount rate**	**Sensitivity analysis**	**Model**
Ara ’07 [76]	Sibutramine + diet and lifestyle	Diet and lifestyle	20–75+ y, BMI ≥ 30	Finland,Germany, UK, Switzerland, CUA	1 year	5 years	5 years	Healthcare	QALY	Literature review	2,149 for Finland, 13,707 for Germany, 10,734 for Switzerland, 11,811 for UK (€/QALY)	2004, 5%, UK (3.5%)	Univariate	DAM
Brennan ’06 [77]	Sibutramine + diet and lifestyle advice	Diet and lifestyle	>40 y, overweight	Germany, CUA	1 year	5 years	5 years	Healthcare	QALY	Literature review	13,706 €/QALY	2003, 5%	Univariate	DAM
Gillies ’08 [84]	Screening for T2DM, screening + lifestyle intervention, screening + drug	No screening	25/45–75 y, BMI > 25, other diabetic risk	UK, CUA	-	-	50 years	Healthcare	QALY	Literature review	14,150 for screening, 6,242 for screening + lifestyle, 7,023 for screening + drug (£/QALY)	2006, 3.50%	Univariate, probabilistic	DAM
Hampp ’08 [81]	Lifestyle intervention, lifestyle intervention + rimonabant	No treatment	≥18 y, BMI > 27 or 30	USA, CEA, CUA	1–2 years	-	5 years	Healthcare	QALY	Three published clinical trials	52,936 US$/QALY for 2 years rimonabant + lifestyle	2006, 3%	Univariate, probabilistic	DAM
Iannazzo ’08 [79]	Orlistat + lifestyle intervention	Lifestyle intervention	≥35 y, BMI > 30	Italy, CUA	4 years	6 years	10 years	Societal	QALY	Single study (RCT)	ICER 75,300 €/QALY	Not mentioned, 4%	Probabilistic	DAM
Prosser ’00 [83]	Low-cholesterol diet, statins	No intervention	35–84 y, LDL ≥ 160 mg/dl	USA, CUA	-	-	30 years	Societal	QALY	Literature review	ICER for diet ranged from 1,900 US$ to 500,000 US$/QALY and statins from 54,000 US$ to 1,400,000 US$ per QALY	1997, 3%	Univariate	DAM
Roux ’06 [82]	Diet, diet + pharmacotherapy, diet + exercise, diet + exercise + behavior modification	Standard care	35 y, BMI ≥ 25	USA, CEA, CUA	6 months	6 months	Lifetime	Healthcare	QALY	Literature review	12,600 US$/QALY for diet + exercise + behavior modification	2001, 3%	Univariate	DAM
van Baal ’08 [80]	Low-calorie diet, orlistat + low-calorie diet	No treatment	20–70 y, BMI ≥ 30	Netherlands, CUA	1 year	-	up to 80 years	Healthcare	QALY	Literature review	ICER 17,900 €/QALY for low-calorie diet and 58,800 €/QALY for orlistat + low-calorie diet	2005, 1.5% to effect & 4.0% to cost	Univariate, probabilistic	DAM
Warren ’04 [78]	Sibutramine + diet and lifestyle	Diet and lifestyle	18–65 y, BMI 27–40	UK and USA, CUA	1 year	5 years	5 years	Healthcare	QALY	Literature review	ICER for sibutramine 4,780 £/QALY	2000, 6% in UK and 3% in USA	Univariate	DAM

Abbreviations: BMI, body mass index; BP, blood pressure; CBA, cost–benefit analysis; CCA, cost-consequence analysis; CEA, cost-effectiveness analysis; CHD, coronary heart disease; CHF, Swiss franc; CUA, cost–utility analysis; CVD, cardiovascular disease; DALY, disability-adjusted life years; DAM, decision analytic model; DPP, Diabetes Prevention Program; DPS, Diabetes Prevention Study; EE, Economic evaluation; GP, general practitioner; ICER, incremental cost-effectiveness ratio; IDPP, Indian diabetes prevention program; IFG, impaired fasting glucose; IGT, impaired glucose tolerance; LDL, low-density lipoprotein; LYG, life years gained; NP, not performed; PA, Physical activity; QALY, quality-adjusted life years; RCT, Randomized controlled trial; SEK, Swedish krona; T2DM; Type 2 diabetes; y, years.

**Table 6.** Characteristics of decision analytic model (DAM).

**First author, year**	**Model**	**Health states in model**	**Population**	**Time horizon**	**Risk factor**	**Effectiveness data**	**Effectiveness measure**	**Methods/Instruments**
Ackermann ’06 [40]	Markov model (CDC)	Nephropathy, neuropathy, retinopathy, coronary heart disease and stroke	DPP participant	Lifetime	From DPP	Single study (DPP)	QALY	QWB
Ara ’07 [76]	Decision tree	CHD, diabetes	Hypothetical	5 years	CHD from Framingham and others from literature	SAT clinical trial and literature review	QALY	SF-36
Bemelmans ’08 [63]	Markov model (RIVM-CDM)	CHD, T2DM, certain cancers, low-back pain, arthritis	Entire Dutch population	Lifetime	Age, body weight, physical activity, disease state, risk factor classes	Two studies from Netherlands	QALY/LYG	Not clear
Booth ’07 [64]	Markov model	11 states: BPG0, BPG1, BPG2, BPG3, CHD, CVE, CHD&CVE, CVE&CHD, CHD death, other death, CVE death	Representative Finnish population	10–40 years	Framingham	National Health Examination Survey	LYG, QALY	15D
Brennan ’06 [77]	Decision tree	CHD, Diabetes	Hypothetical German population of 1,000	5 years	Framingham	Literature review	QALY	SF-36
Brown ’07 [66]	Life table approach	Hypertension, hypercholesterolemia, T2DM, CVD, stroke	Single study population	24 years	Life table Framingham model	Single study (CATCH)	QALY	Not clear
Caro ’04 [43]	Markov model	IGT, NGT, T2DM, death	Hypothetical population of 1,000	10 years	Literature review	DPP, DPS and STOP-NIDDM for acarbose	LYG	-
Colagiuri ’08 [65]	Decision tree	15 health states	Entire Australian population aged 45–74	10 years (2000–2010)	Not clear	DPP, DPS and UKPDS	DALY	-
Dalziel ’07 [60]	4 Markov models	1. Cardiac model: free of further events, minor events, AMI, major events, stroke, and death; 2. Diabetes model: DM, IGT, NGT, death; 3.Fruit & vegetable model: Success, failure, death. 4. BMI model: Normal, overweight, obese, and death	Entire Australian population	20 years (5 years for 2 studies)	-	Literature review	QALY	SF-36, EQ 5D, AqoL, Time tradeoff
Dalziel ’06 [55]	Markov model	3 states: physically active, physically inactive, and dead	Hypothetical cohort (matched with trial population)	Lifetime	Literature review	Single study	QALY	SF-36
Eddy ’05 [44]	Archimedes model	Diabetes, hypertension, asthma, CHF, retinopathy, stroke, nephropathy, neuropathy, death	Hypothetical population (matched with DPP)	5–30 years	-	Literature review	QALY	QWB-SA
Galani ’08 [46]	Markov model	Overweight, hypertension, diabetes, hypercholesterolemia, stroke, CHD	Hypothetical Swiss population of 10,000	65 years (25–85)	Framingham	DPS	QALY & LYG	Not clear
Galani ’07 [47]	Markov model	Overweight, hypertension, diabetes, hypercholesterolemia, stroke, CHD	Hypothetical Swiss population of 10,000	Lifetime	Framingham	DPS	QALY	Not clear
Gillies ’08 [84]	Markov model and decision tree	7 states: NGT, IGT diagnosed, IGT undiagnosed, T2DM (screening detected, clinically detected, undiagnosed)	Hypothetical population starting age 40	50 years	Literature review	Literature review	QALY	EQ 5D
Hampp ’08 [81]	Decision tree	CHD & diabetes, only CHD, only diabetes, no CHD, and no diabetes	Hypothetical population	5 years		Three published clinical trials	QALY	Literature, VAS, TTO
Herman ’05 [38]	Markov model (CDC)	Nephropathy, neuropathy, retinopathy, CHD, and stroke	Hypothetical population	Lifetime	CDC model risk factors	Literature review	QALY	QWB-SA
Hoerger ’07 [41]	Markov model (CDC)	Three modules: screening, prediabetes, and diabetes	Hypothetical population	Up to 75 Years	CDC model risk factors	Literature review	QALY	QWB-SA
Iannazzo ’08 [79]	Markov model	3 states: obese without diabetes, obese with diabetes, and death	Hypothetical Italian population	10 years	Framingham	Single study (RCT)	QALY	Not clear
Icks ’07 [42]	Decision tree	Screening, prediabetes, and diabetes	German population from KORA study	3 years	-	DPP	incidence of diabetes avoided	-
Jacobs- van ’07 [75]	Markov model (RIVM-CDM)	Diabetes, CVDs, cancers, musculoskeletal disease	Dutch population	Lifetime (70 years)	Literature review	Literature review	QALY	Not clear
Lindgren ’07 [48]	Markov model	IGT, MI, stroke, MI 2nd y, stroke 2nd y, T2DM, death	A 60-year-old Swedish cohort	6 years	DPS, UKPDS	DPS	QALY	EQ-5D
Lindgren ’03 [73]	Markov model	10 states: without CVD, 1st and 2nd y of UA, MI, UMI, angina, death	A 60-year-old Swedish cohort	Lifetime (60–109 years)	Framingham	Single study (RCT)	LYG	-
Palmer ’04 [39]	Markov model	IGT, T2DM, deceased	Hypothetical population (matched with DPP)	Lifetime	DPP	DPP and literature review	LYG	-
Prosser ’00 [83]	Markov model (CHD Policy Model)	3 models at the same time (AP, MI, cardiac arrest, coronary revascularization)	Women and men 35–84 years	30 years	HDL, LDL, age group, sex, smoking status, diastolic BP	Literature review	QALY	SF-36
Roux ’06 [82]	Markov model	AP, MI, cardiac arrest	Hypothetical 10,000 obese women	Lifetime	Framingham	Literature review	QALY, LYG	Not clear
Roux ’08 [54]	Markov model (CDC MOVE model)	10 health states, 4 levels of physical activity, CHD, ischemic stroke, T2DM, breast cancer, colon cancer	Hypothetical USA population	40 years	-	Literature review	LYG, QALY	QWB-SA
Salkeld ’97 [70]	Model (Johannesson et al)	CHD (MI, UMI, AP, coronary insufficiency, sudden death), stroke, non-CVD death	Hypothetical population	1 year	Framingham	One Australian trial and literature review	QALY, LYS	TTO
Tice ’01 [61]	Markov model (CHD Policy Model)	3 models (AP, MI, cardiac arrest, coronary revascularization)	Entire US population	10 years	Framingham	Literature review	QALY	TTO
van Baal ’08 [80]	Markov model (RIVM-CDM)	CHD, stroke, diabetes, osteoarthritis, low back pain, some cancers	Entire Dutch population	80 years	-	Literature review	QALY	Person tradeoff
Wang ’03 [68]	Life table approach	-	Single trial population	40 years	Literature review	Single trial (Planet Health) and others	QALY	Not clear
Warren ’ 04 [78]	Decision tree	CHD, diabetes	Hypothetical 1000 population	5 years	Framingham	Literature review	QALY	SF-36

Abbreviations: 15D, 15 dimensions; AMI, acute myocardial infarction; AP, angina pectoris; BP; blood pressure; BPG, blood pressure group; CDC, Centre for Disease Control and Prevention; CHD, coronary heart disease; CHF, coronary heart failure; CVE, cerebrovascular events; DPP, Diabetes Prevention Program; DPS, Diabetes Prevention Study; EQ-5D, Euro Qol 5 Dimension; HDL, high-density lipoprotein cholesterol; IGT, impaired glucose tolerance; LDL, low-density lipoprotein cholesterol; LYG, life years gained; MI, myocardial infarction; NGT, normal glucose tolerance; QALY, quality-adjusted life years; QWB, quality of well being scale; QWB-SA, quality of well being scale—self-administered; RIVM-CDM, RIVM chronic disease model; SF-36, Short Form 36; T2DM, type 2 diabetes mellitus; TTO, time tradeoff; UA, unstable angina; UKPDS, United Kingdom Prospective Diabetes Study; UMI, unrecognized myocardial infarction; VAS, visual analogue scale.

**Table 7.** Uncertainty around long-term effectiveness of lifestyle interventions.

**First author, year**	**Base case Assumption**	**Intervention period**	**Sensitivity analysis**	**Sensitivity analysis assumption**	**Base case result**	**Changes in result due to sensitivity analysis**
Ackermann ’06 [40]	Intervention and effects continued until patients developed disease or died	-	Univariate	Intervention will be only 50% effective	1,288 US$/QALY	Not clear
Ara ’07 [76]	Weight loss regained within 5 years of intervention	1 year	Univariate	Higher and lower rate of weight regain	2,149 €/QALY for Finland,13,707 €/QALY for Germany,10,734 €/QALY for Switzerland, 11,811 €/QALY for UK	14% around the ICER for all countries
Bemelmans ’08 [63]	Effect stops after intervention period	1 year	Univariate	The effect varies 1–4 percentage points	5,700 €/QALY	5,600 €/QALY to 9,900 €/QALY
Brennan ’06 [77]	Weight loss regained within 5 years of intervention	1 year	Univariate	Weight regain equals upper and lower CI, Delay weight regain by 3 months and 6 months	13,706 €/QALY	15,747 and 11,830 for CI, 10,404 and 8,235 for 3 months’ and 6 months’ delay
Caro ’04 [43]	Lifestyle intervention will be 58% effective	5 years	Univariate	Lifestyle intervention will be 30% and 70% effective	ICER 749 CA$/LYG	9,445 CA$/LYG for 30% and “dominant” for 70%
Colagiuri ’08 [65]	The effect will persist as long as intervention continues	10 years	Univariate	Complications reduced to half	50,000 AU$/DALY	Approx. 86,000 AU$/DALY
Dalziel ’06 [55]	Effect returns to baseline at 4 years	3 weeks to 2 years	Univariate, probabilistic	Intervention effect returns to baseline at 1 years, 5 years, 10 years	2,053 NZ$/QALY	10,381 NZ$/QALY (for 1 year), 1,663 NZ$/QALY (for 5 years), 1,160 NZ$/QALY (for 10 years), At 10,000 NZ$ WTP, 97% chance of being cost-effective
Eddy ’05 [44]	The effect will persist as long as the intervention continues		Univariate	20% lower and 20% higher effect on QALY	143,000 from healthcare and 62,600 from societal (US$/QALY)	178,000 and 120,000 from healthcare, 78,000 and 52,000 from societal
Finkelstein ’06 [71]	Effect will persist until death	1 year	Univariate	Effect will persist only 1 year	4,400 US$/LYG	44,500 US$/LYG
Galani ’07 [47]	Weight loss maintained for 6 more years and 4 years to regain the weight. After 10 years the weight reaches the baseline	3 years	Probabilistic	-	64 CHF/QALY for female and - 354 CHF/QALY for male in borderline group	At 1,000 CHF WTP, 99% chance of being cost-effective
Galani ’08 [46]	The weight loss and CVD risk reduction persist for 6 more years and 4 years to regain the weight. After 10 years the weight reaches the baseline	3 years	Probabilistic	-	ICER 4,358 CHF/QALY (Female) and 2,189 CHF/QALY (Male) 30 years old and overweight	At 4000 CHF WTP lifestyle intervention has 45% (Female) and 75% (Male) chance of being cost-effective
Gillies ’08 [84]	Intervention and effects persisted until patients died	-	Univariate, probabilistic	-	6,242 £/QALY	At £20,000 WTP, 99% chance of being cost-effective
Hampp ’08 [81]	Weight loss persists 1 year	1–2 years	Univariate, probabilistic	Weight loss persists 0.5–3 years	52,936 US$/QALY	35,000 (0.5 years) and 62,000 (3 years). At US$ 50,000 WTP 40.2% chance of being cost-effective
Herman ’05 [38]	Effect will persist until participants contract disease	-	Univariate, probabilistic	The effect will decline by 50% and 20%	1,100 US$/QALY	3,102 and 7,886 US$/QALY
Hoerger ’07 [41]	Intervention continued until patients developed disease or died	-	Univariate	The risk reduction from DPP will decline by 20% each year	Strategy one,8,181 US$/QALY; Strategy two, 9,511 US$/QALY	Strategy one, 13,179 US$/QALY; Strategy two, 14,387 US$/QALY
Jacobs-van ’07 [75]	Effect stops after intervention period	5 years	Univariate	No sensitivity analysis in this issue	-	-
Lindgren ’07 [48]	Effect stops after intervention period	4 years	Univariate	Effect of intervention persists for 2 years	2363 €/QALY	Dominant
Lindgren ’03 [73]	Risk reduction effect will persist lifelong (109 y) or the effect will persist only 2 years	6 months	Univariate	-	ICER 127,065 SEK/LYG with declining effect and 141,555 SEK/LYG with remaining effect	-
Palmer ’04 [39]	The effect will persist as long as intervention continues	3 years	Univariate	The effect will persist lifelong	24.56 year improved life expectancy	25.21 year improved life expectancy
Roux ’06 [82]	Long-term maintenance will be 20%		Univariate	Long-term maintenance will be ≤10% and >40%	12,600 US$/QALY	50,000 for 10% and 6,000 for 40% maintenance US$/QALY
Roux ’08 [54]	33% to 50% decline of benefit after intervention	12 months	Univariate, probabilistic	-	ICER 14,286 to 68,557 US$/QALY	At 200,000 WTP, 100% chance of being cost-effective
Salkeld ’97 [70]	Effect stops after intervention period.	1 years	Univariate	Effect will persist 1 year more in high-risk group	ICER 152,128 AU$/QALY for males	ICER 6,589 AU$/QALY
van Baal ’08 [80]	23% of the weight loss achieved after 1 year will be maintained in the long run	1 year	Univariate, probabilistic	50% and 100% weight-loss maintenance in both interventions	ICER 17,900 €/QALY for low-calorie diet and 58,800 €/QALY for orlistat + low-calorie diet	ICER range 8,100–17,800 €/QALY for low-calorie diet and 24,100–18,700 €/QALY for low-calorie diet + orlistat
Warren ’04 [78]	The weight regain to baseline will completed within 50 months for participants and 18 months for placebo group	1 year	Univariate, multivariate	Weight regain equals upper and lower CI	ICER 4,780 £/QALY	4,828 £/QALY and 4,731£/QALY

Abbreviations: CI, confidence interval; ICER, incremental cost-effectiveness ratio; LYG, life years gained; QALY, quality-adjusted life years; WTP, willingness to pay; y, years.
